# Detrimental effects of clothianidin on foraging and dance communication in honey bees

**DOI:** 10.1371/journal.pone.0241134

**Published:** 2020-10-29

**Authors:** Léa Tison, Aron Duer, Vanda Púčiková, Uwe Greggers, Randolf Menzel

**Affiliations:** Institute of Biology-Neurobiology, Free University, Berlin, Germany; University of California San Diego, UNITED STATES

## Abstract

Ongoing losses of pollinators are of significant international concern because of the essential role they have in our ecosystem, agriculture, and economy. Both chemical and non-chemical stressors have been implicated as possible contributors to their decline, but the increasing use of neonicotinoid insecticides has recently emerged as particularly concerning. In this study, honey bees were exposed orally to sublethal doses of the neonicotinoid clothianidin in the field in order to assess its effects on the foraging behavior, homing success, and dance communication. The foraging span and foraging activity at the contaminated feeder decreased significantly due to chronic exposure at field-realistic concentrations. Electrostatic field of dancing bees was measured and it was revealed that the number of waggle runs, the fanning time and the number of stop signals were significantly lower in the exposed colony. No difference was found in the homing success and the flight duration between control and treated bees released at a novel location within the explored area. However, a negative effect of the ambient temperature, and an influence of the location of the trained feeder was found. Finally, the residues of clothianidin accumulated in the abdomens of exposed foraging bees over time. These results show the adverse effects of a chronic exposure to sublethal doses of clothianidin on foraging and dance communication in honey bees.

## 1. Introduction

Pollinating insects contribute significantly to agricultural productivity and the importance of their conservation is no longer up for debate [1, 2]. The prevalent use of pesticides in crop protection and especially the extensive use of neonicotinoids as a prophylactic measure in agriculture poses a threat to pollinating insects [3–6] and led to the ban of 3 neonicotinoids for outdoor use in Europe [7, 8] Indeed, sublethal doses of neonicotinoids were already shown to compromise a large range of behaviors [9–16] and survival in honey bees [6, 17].

Neonicotinoids act as agonists on nAChRs opening cation channels [18] located in the central nervous system of insects. Their agonistic action induces continuous excitation of the post-synaptic membrane, producing discharges leading to cell energy exhaustion, paralysis and death [19]. Clothianidin is a neonicotinoid insecticide formulated to act upon sucking and chewing pest insects. It is mostly applied as a seed coating but also applied as foliar spray or applied to soil for a variety of crops. Clothianidin is a component of several commercial insecticides, but is also the metabolite of thiamethoxam, so clothianidin can be the result of direct exposure or metabolite exposure [20].

At sublethal doses, several laboratory and field studies have shown negative effects of clothianidin in honey bees and other bee species [11, 13, 21–23]. Clothianidin was also shown to exert an immunosuppressive action [22, 24], which further exacerbates the negative impact that viral pathogens and parasites have on honeybee defense barriers [25, 26].

However, the effects of clothianidin on individual bees and on whole colonies have been revealed to be highly variable and a source of debate [27–32], possibly indicating that in natural conditions, honey bees at the colony level might be more robust to the effects of clothianidin [33]. Testing single bees in natural conditions does not predict the survival of the colony in the long term but represents well the conditions faced by bees in nature since honey bees forage as single animals.

Because of the systemic properties of neonicotinoids, insects can be exposed chronically and acutely in the field. Whereas residues in pollen and nectar result most of the time in chronic exposure of forager bees, residues in water puddles, guttation drops, and in dust drift can lead to acute exposure of foragers or honey bee colonies [34–36]. The intake from nectar and pollen residues from oilseed rape, at the lowest and highest maximal application rate was estimated by the EFSA [37] to be 4.27 ng and 13.65 ng per forager bee in one day respectively, both estimations being above the endpoint of acute oral toxicity for clothianidin (LD50_48h_ = 3.7 ng/bee). Our lab and field experiments were conducted with field-realistic concentrations or doses, similar or lower to the EFSA [37] estimations (S1 Table) and to the doses used in several other studies [11, 13, 23]. Reported values of the maximum amounts of clothianidin residues found in the nectar of treated crops vary from 1 to 14 ppb with the average values ranging from 0.3 to 5.4 ppb [30, 35, 38, 39].

In 2008 in Germany, the registration of clothianidin for use on seed corn was revoked after an incident caused by the abrasion of clothianidin-treated seeds during sowing that resulted in the death of millions of nearby honey bees [40]. In 2013, The EFSA (European Food Safety Authority) identified a risk of clothianidin to bees exposed to contaminated dusts and residues in nectar and pollen from rape [37]. In February 2018, the EFSA has evaluated data collected in an open call for the review of the 2013 restrictions and the Commission and the Member States confirmed the already identified risks of neonicotinoids and fipronil for outdoor uses [8]. Member States endorsed the Commission's proposals to completely ban the outdoor uses of the three active substances clothianidin, imidacloprid and thiamethoxam.

Effective foraging in honeybees requires the coordination of several cognitive functions including navigation using a sun compass, learning of the spatial relations between landmarks and social communication by the waggle dance and the various response signals from the dance followers [41]. The common bases of all these functions is learning by experience and a symbolic form of communication. Since even rather simple forms of learning like odor conditioning was found to be impaired by clothianidin [42], it is likely that these demanding forms of learning and behavioral control might also be affected by clothianidin. In this study, honey bee foragers (*Apis mellifera*) were chronically exposed at feeders in a field to 4.5 and 9 ppb clothianidin in sucrose solution in order to investigate its effects on foraging behavior, dance communication and homing success. Previous study has shown that thiacloprid, another neonicotinoid, had dramatic consequences on these behaviors including reduction of waggle dance communication [14]. Using electrostatic field recordings [43], it was found that the colony whose foragers were exposed to a contaminated feeder with thiacloprid performed fewer dances than the control colony [14]. In this study, a new set-up for electrostatic field recordings was used, allowing to distinguish between fanning, waggle dancing and stop signals. Until now, no study has investigated the effects of clothianidin on such a large range of behaviors by exposing animals to chronic concentrations.

## 2. Material and methods

### 2.1. Clothianidin solutions

A stock solution of 0.25 g/L was made by diluting 10 mg clothianidin ((*E*)-1-(2-chloro-1,3-thiazol-5-ylméthyl)-3-méthyl-2-nitroguanidine, Sigma-Aldrich) in 1 mL acetone (≥99.9%, Sigma-Aldrich) plus 39 mL distilled water. Acetone was chosen as the solvent following the EPPO guidelines [44]. The control group was fed sucrose solution without acetone as it was demonstrated in previous studies that acetone at concentrations much higher than the ones used in this study (0.00005 and 0.0001% acetone in the solutions used in the feeders) had no effect on sucrose perception [14, 15] nor on behavior [11]. Each clothianidin sucrose solution used in the field (1 M, 0.5 M or 0.25 M) was generated at two different levels of ppb (4.5 and 9 ppb) and freshly made every morning from the stock solution. The sucrose solution concentrations are based upon expected environmental exposure [37], at a level known to have sublethal effects on bees [13]. The concentration of the solutions used were confirmed by LC-MS/MS.

### 2.2. Experimental design

The experimental area was a highly structured agricultural landscape (trees and bushes, pathways, creek, grass fields, etc.) nearby Großseelheim, Germany. Permission of the owner was given for the use of private land and activities and the field study did not involve endangered or protected species. Two colonies housed in two observation hives (W.Seip, Bienenzuchtgerätefabrik) were put up on two opposite sides of a cabin (50°48'51.9"N). Each colony of *Apis mellifera carnica* was equipped with one comb (Deutsch Normal Mass) of sealed brood plus newborn bees and one comb of food originating from the same honey bee colony. The queens were generously provided by the Bieneninstitut Kirchhain and the bees from a local beekeeper. Queens were sisters, open mated, aged 1 year old, and derived from selected breeder colonies of the carnica breeding population of the institute.

#### 2.2.1. Training to the feeders

The experimental set-up was the same as in the Tison *et al*. [14] study except for the harmonic radar which was not used in this study due to technical issues. Two feeders (F1 and F2) were separated by an angle of 90° and established 350 meters northeast and 340 southeast from the cabin respectively. The release site was located 780 meters east of the cabin. A group of foragers from the control colony was trained to the control feeder and a group of foragers from the treated colony was treated to the contaminated feeder. Each bee visiting the feeder was caught after feeding on the sucrose solution and put in a marking catcher between a net and a foam to be marked individually without cooling with a number tag on the thorax. Note was taken about the number and identity of bees visiting the control and contaminated feeders as well as the amount of sucrose solution consumed every day. The origin of each newly marked bee was also controlled at the respective hive entrance. The foraging span of bees visiting the control or contaminated feeder was calculated by counting the number of days between the day a bee was caught and marked at its feeder and the last day a bee was seen visiting its feeder, ensuring also that the bee visited the feeder every day between the first and last day.

Each feeder was placed in a little wooden box to allow for counting the entrances and exits of foragers with a retro-reflective sensor (Baumer GmbH). In order to regulate the traffic, the concentration of the sucrose solution at each feeder was adjusted during the day following evaluation by the experimenter of the number of trained foragers visiting the feeder. Dance recruitment was induced at the same time (1400–1600 hours) at both feeders, once a day, on 24 different days by first halving the sucrose concentration at both feeders for one hour and then increasing it twofold for another hour [45].

Both control and treated bees foraged first on uncontaminated sucrose solutions for 7 days. Experiment 1 started seven days later (July. 31) when one group of bees (treated group) began foraging on a sucrose solution containing clothianidin (4.5 ppb), and the other group (control group) began foraging at a feeder containing only sucrose solution. One week later (Aug. 7), the concentration of clothianidin was then raised at the treated feeder to 9 ppb for 11 days. The exposure to clothianidin was interrupted between Aug. 14 and Aug. 22 because the location of the control and treated feeders was switched and bees were trained to the new locations for experiment 2. The feeders´ locations were exchanged in order to exclude any possible landscape effect related to the feeders’ position. After exchanging the location of the feeders, the two groups of foragers continued feeding at their respective feeder during 13 days until Sep. 3 (experiment 2). A timeline of the experimental design is visible in Fig 3.

#### 2.2.2. Homing experiment

Colonies were settled in the field for at least a week before the homing experiments started. After a certain number of days foraging at the feeders, single bees were caught on departure at their respective feeder after they had freely drunk a 1 M sucrose solution containing 4.5 or 9 ppb clothianidin (treated bees) or not (control). They were kept in the dark for 50 min while they were transported into a ventilated glass vial to the release site. Bees from both feeders were tested each day and the time (between 1200 and 1800 hours), temperature (16–27°C) and wind (< 15 km/h) were noted. No release was made when the sky was evaluated too cloudy or totally overcast, nor when it was raining so homing foragers could use celestial cues. Each bee was released only once. A 120 min waiting time was set for each released bee before stopping to look at the entrance of the hive. Bees that did not return to the hive within 120 min after being released and not seen at the feeder or at the hive entrance on the same or the following days were considered to have died in the field.

#### 2.2.3. Electrostatic field measurements

**Fig 1:**
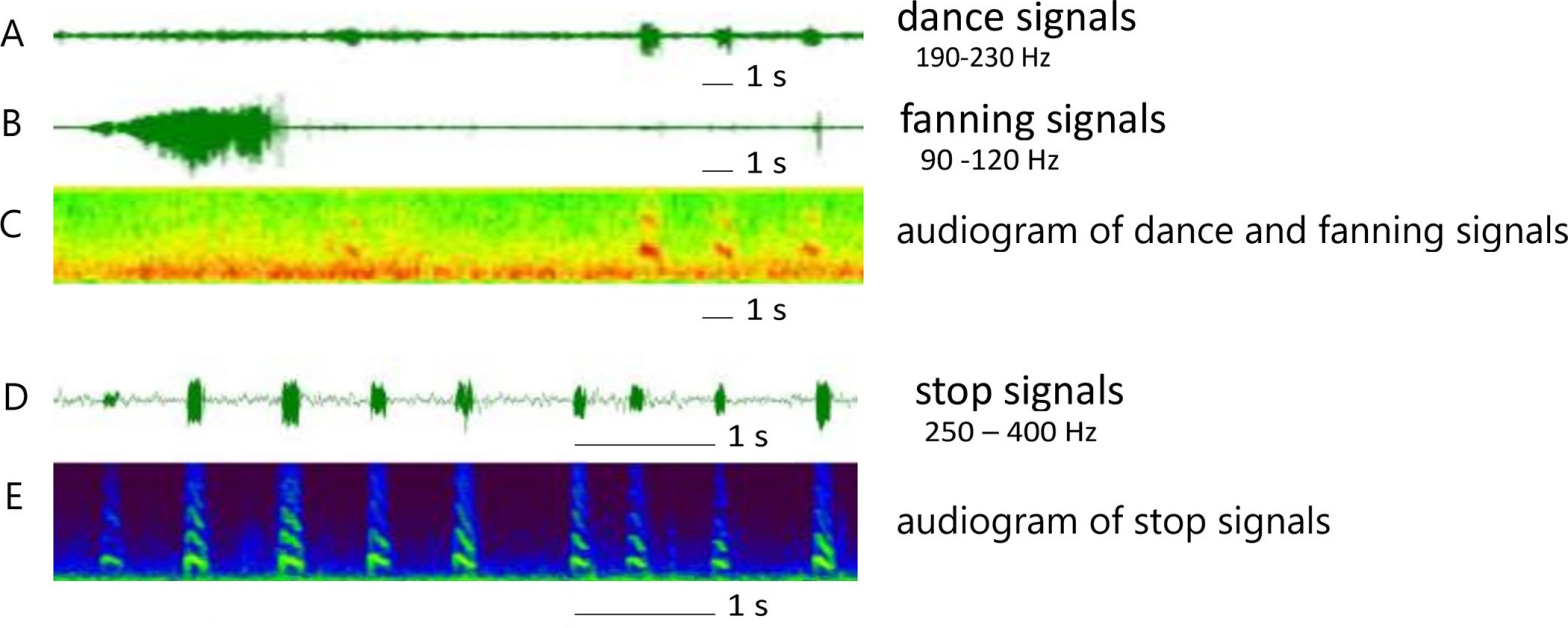
Electrostatic field (ESF) signals of the three behaviors, waggle dancing, fanning and stop signals. **A to C**: ESF recording of waggle dances and fanning behavior. Signals of the waggle dance were characterized by a low frequency band (10–25 Hz) and a high frequency band (190–230 Hz). The low frequency band is hidden behind the high frequency band in row **A**, and can be resolved in the audiogram (row **C**) by expanding the low frequencies. Fanning behavior usually lasted > 10 s and was characterized by frequencies in the 90–120 Hz band (rows **B** and **C**). Stop signals were short pulses (< 0.5 s) with frequencies between 250 and 400 Hz (rows **D** and **E**). Multiple harmonics and temporal dynamics characterize these short pulses.

The electrostatic fields emanating from the body of the bees were recorded at the same time during dance induction in both control and treated hives during experiment 2 (9 ppb clothianidin). The method of recording the time modulated electrostatic fields of dancing bees has been described in detail in Greggers *et al*. [43]. In short, the sensors for electrostatic fields were built by removing the membrane of ordinary miniature microphone capsules, which were connected through a trim potentiometer (100 kOhm) to an audio amplifier (Presonus Audiobox 1818 vsl, LA-70802, USA) and driven by an external voltage of 12 V supplied by a rechargeable battery (Hacker Modellbau, D-84030). Four sensors were placed above the dance area behind a 4 mm Perspex plate at a distance of 5 cm to each other on both sides of the lower comb with sealed brood (total area recorded 12 x 12 cm). The amplifier getting input from all sensors was connected via USB to a computer where the amplifiers' software (Presonus StudioOne, LA-70809, USA) recorded the data. The potentiometers were used to set the signals of the different sensors to the same level using a test stimulus. A metal mesh built around the whole observation hive functioned as a faraday cage thus reducing external electric noise. The data were stored as a wav-file for each electrode with 44.1 kHz sample rate and 18 bit depth. The movements of the bee body or parts of it (e.g. the wings) led to emanating electrostatic fields with characteristic time courses and frequency components [43] (Fig 1). The signal strength and the characteristic waveforms including the composition of the frequencies and their harmonics did not differ between bees in the control and the treated colony. Only the occurrence changed. Therefore, the same settings were used to identify the different signals in the two colonies. Waggle dancing led to rhythmic pulses of low (10–25 Hz) and high frequencies (190–230 Hz). Fanning was characterized by long lasting and stable signals of 90–120 Hz, and stop signals were short pulses (< 500 ms) of frequencies > 250 Hz often combined with pronounced harmonics. These signals could be distinguished and counted by custom-made analyzing programs developed in our lab with Python (version 2.7, Wilmington, Delaware, USA). The publication of the code is forthcoming (in prep.).

### 2.3. Clothianidin residue analysis

#### 2.3.1. Preparation of the bee samples

Bees were caught at their feeder after foraging for 1 to 2, 3 to 6, or more than 7 days and after they had filled their crop with a 1 M sucrose solution contaminated with 9 ppb clothianidin or not (control). In order to keep the collected bees calm, they were kept in the dark for 50 minutes before being killed by chilling and put into a -20° C deep-freezer.

Unmarked forager bees were collected at the entrance of the treated and control hives when flying out on a foraging trip in order to assess the in-hive contamination of foragers not visiting the feeders but exposed indirectly to clothianidin inside the hive via the stored food. All of the collected bees were cut into 3 parts, head, alitrunk and gaster (the terms thorax and abdomen respectively will be used for convenience). The legs and wings were cut off. Samples from the same foraging groups were pooled (usually 10 body parts in each tube) and weighed. Surrogate solution (25 μl, acetamiprid-d3) and acetone (5 ml) were added to each sample, then homogenized with a disperser during three minutes and centrifuged (Megafuge 165 Heraeus with tx 400 rotor, 10 min at 3000 rpm). After centrifugation, 4 ml of supernatant was carefully removed and left to dry in a metal block thermostat under a gentle stream of nitrogen. Water methanol (950 μl, 1/1, v/v) and 50 μl internal standard solution containing clothianidin-d3 in methanol water (1/1, v/v, 65 pg/μl) were added to the dry extract. Samples were then mixed using an ultrasonic liquid mixer (Elma, Transsonic T 460 / H) and put into the freezer (-18°C) overnight.

#### 2.3.2. Identification and quantification of clothianidin residues

Samples were filtered cold (Phenex™-RC 15mm Syringe Filters 0.2 μm) before proceeding with the identification and quantification of clothianidin by LC-MS/MS. The LC-MS/MS system used was a UltiMateR 3000 RS HPLC (Dionex Corporation, Sunnyvale, USA) coupled to a mass spectrometer QTRAP® 5500 (AB SCIEX, Framingham, USA) equipped with an electrospray ionization (ESI) source. For more details about the method, please see S3 Table.

Clothianidin was identified by its retention time and two Multiple Reaction Monitoring (MRM) transitions. The residues in the samples were measured using matrix standards (concentrations: 0.1, 0.5, 1, 5, 10, 25, 50 pg μL^-1^). The quantification was carried out by the internal standard method. The value given for each sample represents the average of double-injections. See S2 Table for recoveries, limit of detection (LOD) and limit of quantification (LOQ). These values were determined during the method validation. Frozen samples of contaminated sucrose solutions were sampled and analyzed by LC-MS/MS. LOD = 0.05 pg μL^-1^ and LOQ = 0.1 pg μL^-1^.

### 2.4. Statistical analysis

For the statistical analysis of the data, Prism 5 and 6 were used and the software R (version 3.3.2) with packages lme4 [46], aod [47], emmeans [48], survival [49] and multcomp [50]. The normality of the data was tested using the D'Agostino-Pearson omnibus test. If the data were consistent with being normally distributed, a paired/unpaired t-test or an analysis of variances with Tukey’s post-hoc tests was used. Otherwise non-parametric tests were performed (Mann-Whitney test). The Fischer’s Exact Test was used to compare proportions. For the sucrose consumption, the visits to the feeders, and the electrostatical field results, fixed factors included in the model were treatment or dose and experiment in interaction. Days of recording and sucrose concentration were used as covariables. Comparisons of Estimated Marginal Means (EMMeans) were performed as post-hoc tests, with Tukey-adjusted *p*-values.

The survival analysis was conducted using censored Kaplan Meier Log-Rank in R and the influence of multiple variables was investigated using a Cox-regression model. Several models (with or without interactions between factors) were tested and the best was selected using AIC (Akaike information criterion). All models were validated by assessing normal Q-Q plots and residual versus fitted data plots. This was followed by Overall Likelihood Ratio Tests and Tukey’s post-hoc tests. *Chi-square* tests were used to compare the mortality and US-tests rates between the doses. Comparisons in which P < 0.05 were considered significant. The numbers of bees tested for each experiment and test groups or the number of recording days are indicated in the legends of the figures and in the text.

## 3. Results

### 3.1. Foraging behavior

**Fig 2:**
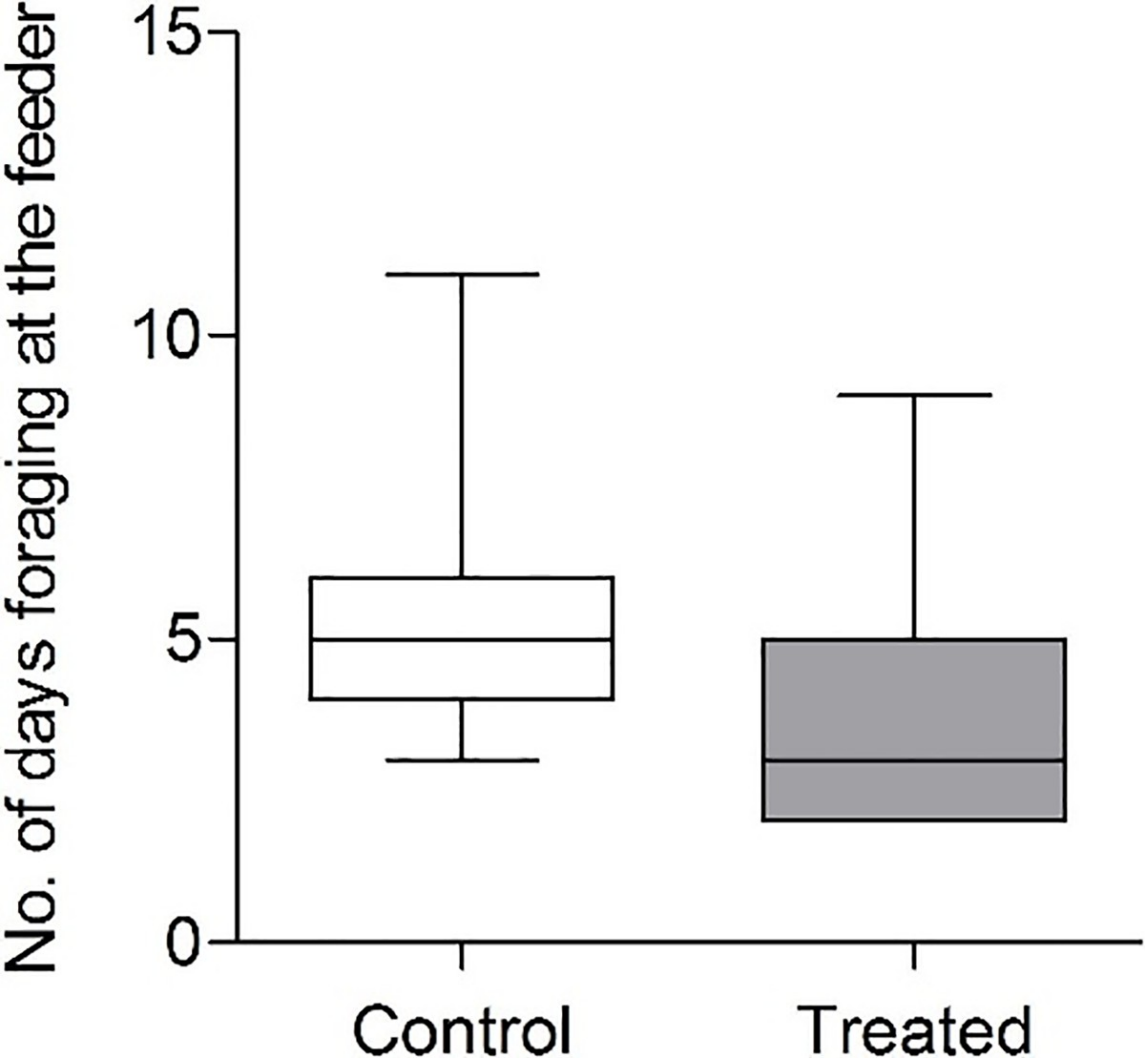
Foraging span of trained bees at the control and treated feeders (Means ± S.E.M, *n*_control_ = 56, *n*_treated_ = 81). The treated feeder contained 4.5 ppb or 9 ppb clothianidin in sucrose solution and the control feeder sucrose solution only. The foraging span was significantly shorter for the treated group of bees (Mann Whitney, P < 0.001).

Because exposure to 9 ppb followed directly exposure to 4.5 ppb and bees were then often exposed to both concentrations, the data on the effects of treatment were calculated for the 4.5 and 9 ppb concentrations together. Treated bees were found to forage on average 2 days less (Fig 2, 2–9 days, median = 3 days) than control bees (3–11 days, median = 5 days) over the same period of time (Fig 2, Mann Whitney, P < 0.001).

Next, the amount of sucrose solution collected at both feeders throughout the summer was evaluated and no significant difference between the control feeder and the feeder contaminated with 4.5 ppb or 9 ppb clothianidin was found (ANOVA of lm, P = 0.41). During experiment 2, the control bees consumed on average 1.3 times more sugar solution per day than treated bees but the difference was not significant (Tukey, P = 0.35). Treated bees exposed to clothianidin during experiment 1 did not collect less sucrose solution than the control bees (Tukey, P = 0.98).

The average amount of clothianidin estimated to be collected per bee and per day is related to the amount of sucrose solution collected at the treated feeder and the concentration of the solution. Bees exposed to 4.5 ppb clothianidin collected on average (± S.E.M) 12.95 ± 0.45 ng per day and per individual, and bees exposed to 9 ppb during the first experiment collected 12.90 ± 1.21 ng clothianidin per day and per individual. However, bees exposed to 9 ppb during the first experiment collected on average 20 ml less sucrose per day than bees exposed to 4.5 ppb. Bees exposed to 9 ppb during experiment 2 foraged less than during experiment 1. They collected half as much sucrose solution than bees exposed to the same concentration during experiment 1. The exposure per bee was found higher but not statistically different (Tukey, P = 0.90) in the second experiment (14.32 ± S.E.M 1.62 ng) than in the first experiment (12.90 ± S.E.M 1.21 ng).

**Fig 3:**
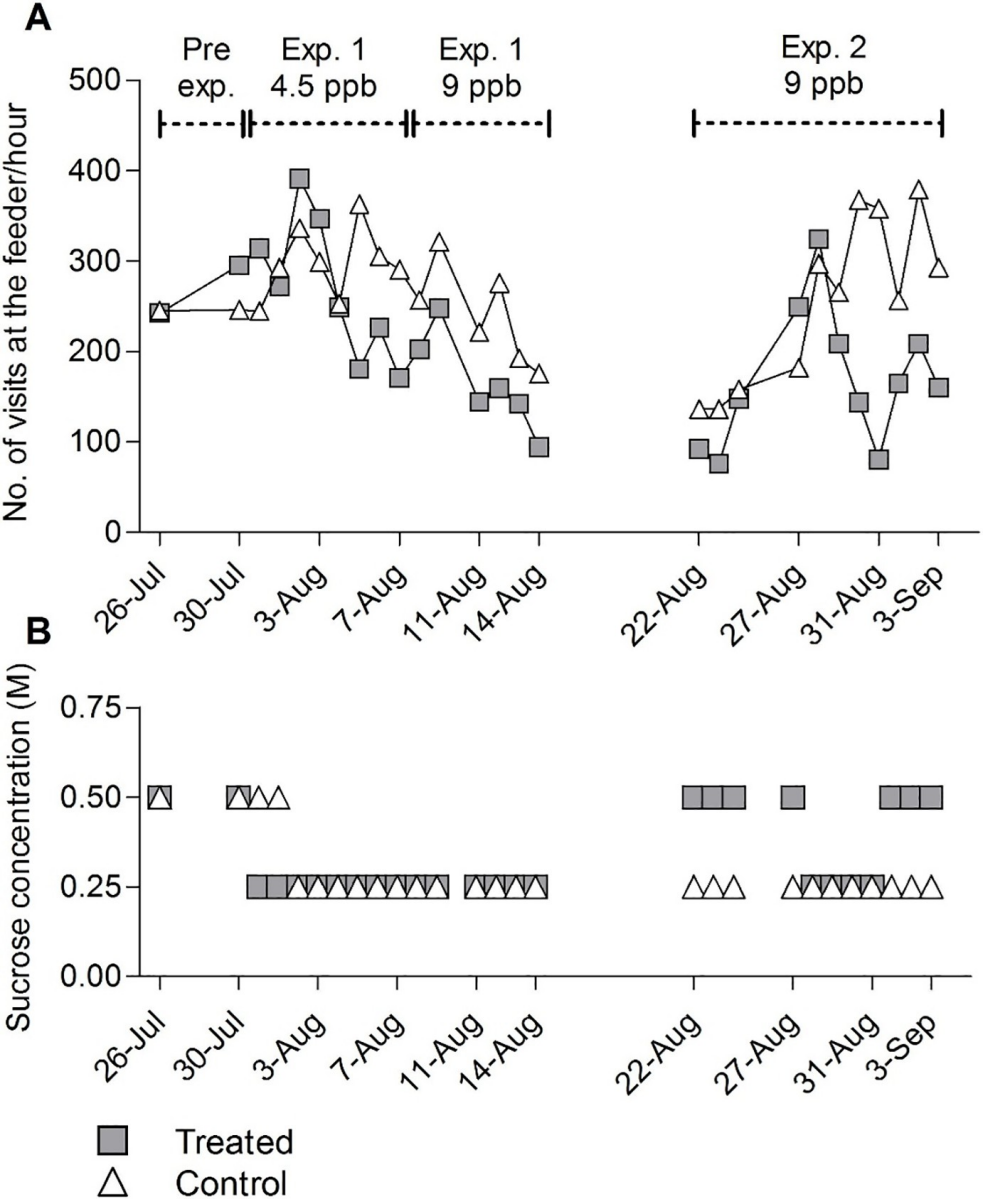
Foraging activity and required sucrose concentrations at the control and treated feeders. (A) Number of visits per hour recorded on the same days (*n* = 27 days) during the pre-exposure time in experiment 1 (4.5 ppb and 9 ppb) and experiment 2 (9 ppb) at both control (triangles) and treated feeders (squares). The foraging activity of the treated bees is significantly reduced by exposure to clothianidin (ANOVA of lm, P < 0.05). (B) Sucrose concentrations used in order to keep a similar number of foragers coming regularly to the control and treated feeders. The same or higher concentrations of sucrose solution were usually used at the treated feeder.

Interestingly, the average number of bees foraging at the control feeder remained unchanged during experiment 1 and 2 for the three groups (4.5 ppb experiment 1, 9 ppb experiment 1 and 9 ppb experiment 2) (S1 Table) whereas the average number of bees foraging at the treated feeder decreased in the three groups from 50 to 40 and then to 27, even though higher concentrations of sucrose were used in order to motivate them to visit their feeder (Fig 3B).

Treated bees performed on average in the three groups 1.2, 1.5 and 1.7 times fewer foraging trips per day than control bees (S1 Table: experiment 1: 4.5 ppb and 9 ppb, experiment 2: 9 ppb). It was estimated that a bee collected on average 0.28 ng clothianidin (about 56 μl of solution of 4.5 ppb solution) and 0.45 ng (about 45 μl of 9 ppb solution) in experiment 1, and 0.53 ng (about 53 μl of 9 ppb solution) in experiment 2 on one trip using the average number of trips per bee and day and the amount of clothianidin collected per bee and day at the respective feeder (S1 Table).

Thus reduced sugar consumption was linked to reduced visitation rates of forager bees at the contaminated feeder. Treated bees visited their feeder less frequently than the control bees on the same days (Fig 3A, ANOVA of lm, P < 0.05). Similar or higher sucrose concentrations were needed at the contaminated feeder in order to keep the bees visiting the feeder (Fig 3B, median control and treated = 0.25 M).

As described in the Methods section, exposure to clothianidin followed a week of training at the uncontaminated feeder (pre-exposure). This allowed us to directly compare the visitation rate by the uptake of the pesticide.

The number of visits per hour was the same or higher during this pre-exposure than when the sucrose solution contained 9 ppb. A linear model with the variables treatment and experiment in interaction was applied to the data of experiments 1 and 2. The model revealed significant effect of the treatment and the day of recording. Fig 3A shows that the treated feeder was on average 33% less frequently visited than the control feeder (ANOVA of lm, P < 0.05). During experiment 1, the number of visits per hour was statistically different between the control and the treated feeder (Tukey, P < 0.05). The visitation at the feeders between August 15 and August 21 was not recorded as it was the period during which the positions of the feeders were switched and bees were trained to their new feeding location. Only on August 27 and 28, the treated feeder was visited more than the control feeder, but the sucrose concentration in the feeding solution was 0.5 M and in the control feeder only 0.25 M. Every other day during the experiment 2 the treated feeder was visited on average 34% less than the control feeder (Fig 3A, Tukey, P = 0.96) even though the sucrose concentration at the treated feeder was more than half of the time higher than at the control feeder (median concentration treated: 0.5M; control: 0.25M).

**Fig 4:**
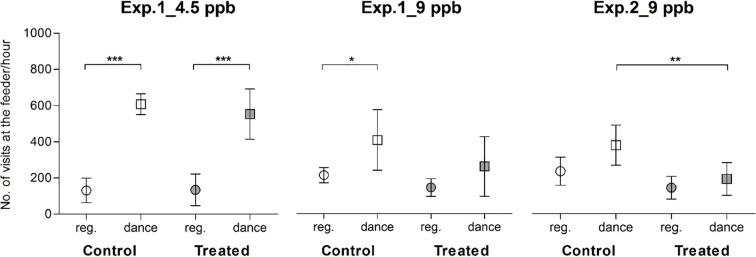
Number of visits per hour performed collectively by the trained bees from the control and contaminated feeder with clothianidin. Mean (± 95% confidence limits) number of visits per hour recorded on the same days at both feeders during regular foraging (“reg.”, circles) and during dance induction (“dance”, squares). Dances were induced at the same time at both feeders on *n* = 7 days in exp. 1, 4.5 ppb, *n* = 6 days in exp.1, 9 ppb and *n* = 11 days in exp. 2, 9 ppb. Both control (unfilled marks) and treated bees (filled marks) with 4.5 ppb increased their number of visits to the feeders in exp. 1. With 9 ppb in exp. 1, only control bees significantly increased the number of visits per hour at their feeder. In exp. 2 (*n days* = 6, 9 ppb), the number of visits per hour was significantly different at the control and treated feeders. Stars indicate the results of the Tukey post-hoc tests after ANOVA: *P < 0.05, **P < 0.01, *** P < 0.001.

Recruitment of foragers via the waggle dance can be induced by raising the sucrose concentration at the feeder [45]. The sucrose concentration in the feeding solutions during the dance induction in experiment 1 was most of the time the same at both feeders (0.5 M) since the regular sucrose concentration was also similar (Fig 3B, 0.25 M). However, during experiment 2, the sucrose concentration during dance induction was more than half of the time higher at the treated feeder. The regular foraging activity and the foraging activity during dance induction on the same days at both feeders and both experiments (1 and 2) for the clothianidin concentrations 4.5 and 9 ppb, were extracted from the data presented above and analyzed with ANOVA and Tukey post-hoc tests. Fig 4 gives the data for both control (unfilled marks) and treated bees (filled marks) with 4.5 ppb showing equal visitations at the feeder in experiment 1 (Fig 4, Tukey, P < 0.001). However, visitations to the feeder were significantly lower for treated bees exposed to 9 ppb in experiment 1 (Fig 4, Tukey, P < 0.05). The visitation rate at the control feeder was 36% higher than at the treated feeder during dance induction in experiment 1. In experiment 2, visitation rate increased by 38% at the control feeder during dance induction, whereas no increase was found at the contaminated feeder (9 ppb) although this effect is not statistically significant. However, the change in visitation (49% increase at the control feeder as compared to the contaminated feeder) in experiment 2 is significant (Fig 4, Tukey, P < 0.01). Interestingly the visitation rate at both feeders decreased during regular foraging and dance induction throughout the summer.

### 3.2. Social signals

#### 3.2.1. Fanning behavior

**Fig 5:**
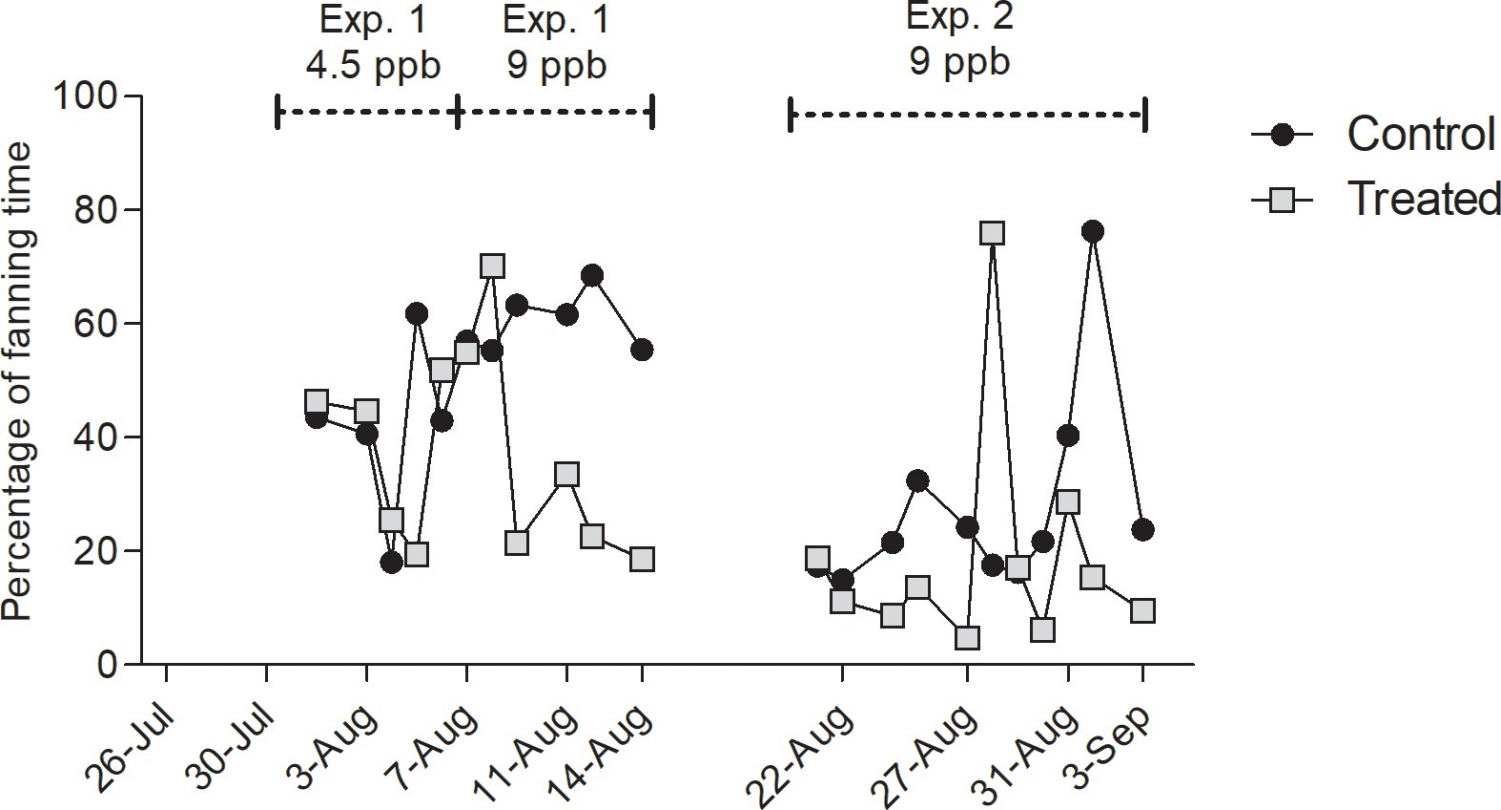
Percentage of the recording with identified fanning behavior. Electrostatic fields emanating from dancing bees were recorded on *n* = 22 days during dance induction, in experiment 1 (4.5 ppb) from Aug. 1 to Aug. 7 and from Aug. 7 to Aug. 14 (9 ppb), and in experiment 2 (9 ppb) from Aug. 22 to Sep. 3 in both control (triangles) and treated hives (squares) at the same time. The percentage of fanning time was significantly higher in the control colony (ANOVA of lm, P = 0.05). The line break represents the one-week off treatment and off recording during which the location of the feeders was switched.

Among 22 days of electrostatic field recordings during dance induction, the percentage of fanning time in the control colony was higher (Fig 5, Mean ± S.E.M = 39.75% ± 4.25) than in the treated colony (Mean ± S.E.M = 28.11% ± 4.37)). The model revealed significant effect of the treatment (ANOVA, P = 0.05) and the experiment (P = 0.001). Indeed, control bees expressed significantly less fanning behavior during experiment 2 than during experiment 1 (Tukey, P < 0.05)

#### 3.2.2. Waggle runs

**Fig 6:**
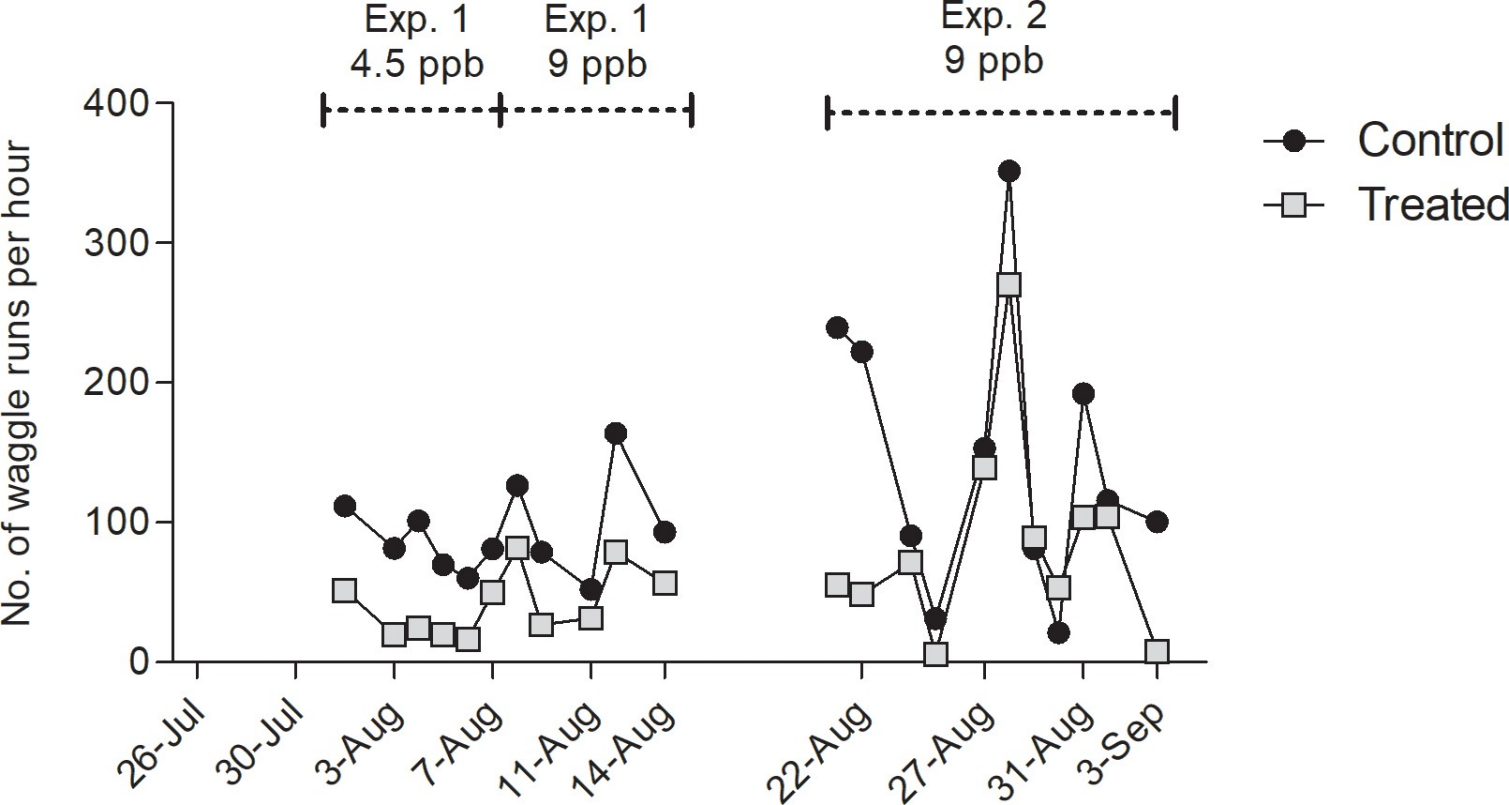
Number of identified waggle runs per hour. Electrostatic fields emanating from dancing bees were recorded on *n* = 22 days during dance induction, in experiment 1 (4.5 ppb) from Aug. 1 to Aug. 7 and from Aug. 7 to Aug. 14 (9 ppb), and in experiment 2 (9 ppb) from Aug. 22 to Sep. 3, in both control (triangles) and treated hives (squares) at the same time. Significantly higher amounts of waggle runs were detected in the control colony (ANOVA of lm, P < 0.0001). The line break represents the one-week off treatment and off recording during which the location of the feeders was switched.

From the very first day of the recordings on Day 2 of treatment with 9 ppb, more waggle runs were detected in the control colony (Fig 6, Mean waggle runs per hour ± S.E.M = 119.1 ± 16.36 in the control colony and 63.92 ± 12.33 in the treated colony). After the break, many more dances happened in the control colony during three days and then the difference between the two groups faded. The model revealed significant effect of the treatment (ANOVA of lm, P < 0.0001). The difference in the number of waggle runs was different between control and treated bees in experiment 1 (Tukey, P < 0.05) and in experiment 2 (Tukey, P < 0.01). The model also revealed significant effect of the experiment (P < 0.0001) since the number of waggle runs differed significantly between control bees from experiment 1 and 2 (Tukey, P < 0.05) and between treated bees from experiment 1 and 2 (Tukey, P = 0.05).

#### 3.2.3. Stop signals

**Fig 7:**
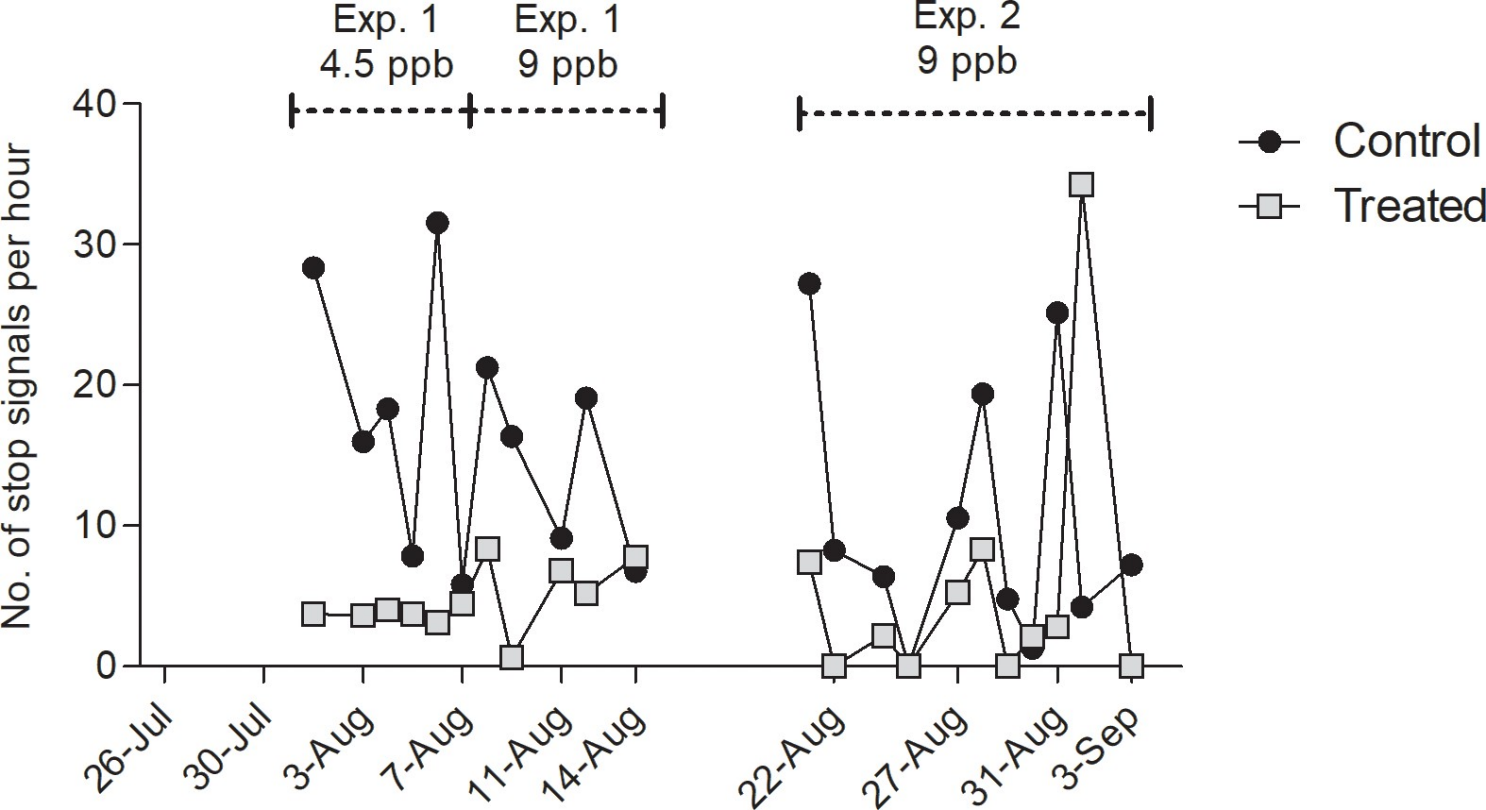
Number of identified stop signals per hour. Electrostatic fields emanating from dancing bees were recorded on *n* = 22 days during dance induction, in experiment 1 (4.5 ppb) from Aug. 1 to Aug. 7 and from Aug. 7 to Aug. 14 (9 ppb), and in experiment 2 (9 ppb) from Aug. 22 to Sep. 3, in both control (triangles) and treated hives (squares) at the same time. Significantly more stop signals were detected in the control colony than in the treated colony (ANOVA of lm, P < 0.01). The line break represents the one-week off treatment and off recording during which the location of the feeders was switched.

Except for the second last day of the experiment, the number of stop signals per hour never exceeded 10 for the treated colony. The Mean number of stop signals was found much higher in the control colony (Fig 7, Mean ± S.E.M = 13.4 ± 1.99) in comparison with the treated colony (Mean ± S.E.M = 5.17 ± 1.5). The effect of treatment was revealed significantly different by the model (ANOVA, P < 0,01, Tukey C1 *vs* T1, P < 0.05) whereas the experiment had no effect (P = 0.32).

### 3.3. Residue analysis

Bees visiting the control and contaminated feeders were caught at their departure from the feeder, immediately after drinking some sucrose solution containing 9 ppb clothianidin.

**Fig 8:**
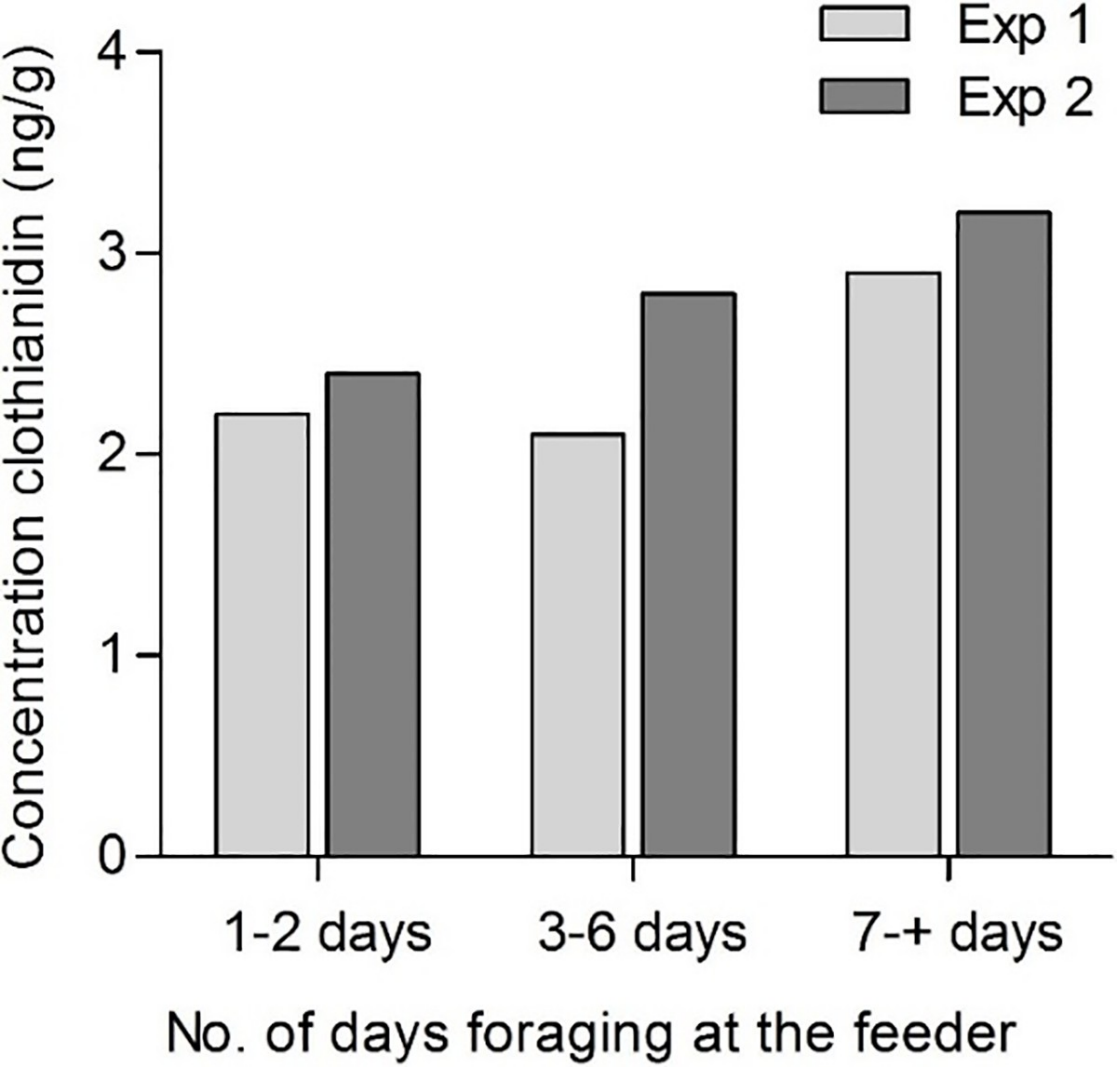
Residues of clothianidin (ng/g) detected in the bees caught at the contaminated feeder with 9 ppb clothianidin. Bees from Exp. 1 were collected on Aug. 16 and bees from Exp. 2 on Sept. 4. Collected bees were grouped (*n* = 10 bees) according to the number of days they foraged at their feeder before analysis.

In experiment 1 the residues ranged between 2.1 ng/g and 2.9 ng/g and in experiment 2 from 2.4 ng/g to 3.2 ng/g depending on the number of days bees foraged at the feeder before being caught for analysis (Fig 8). Clothianidin residues were detectable only in the bee abdomens except in experiment 2, during which clothianidin was detected also in the heads of bees, which foraged 3–6 days at the contaminated feeder (S2 Table).

Residues of clothianidin could not be detected in any of the control samples collected at the feeder or at the hive entrance throughout the summer.

In bees caught at the hive entrance, clothianidin residues were detected only in the abdomens of bees collected after 30 days of treatment (S2 Table, 2.80 ng/g). Divided by the number of bees in this sample, clothianidin residues amount to 0.23 ng of clothianidin per bee.

Clothianidin residues in honey and wax from the control and treated hives were not present in the samples or were under the limit of detection of the LC-MS/MS method used here and thus not presented.

### 3.4. Homing success

Navigation requires the integration of multisensory cues and the retrieval of appropriate memory about the landscape structure. The two groups of bees (control, treated) were trained to two different feeders. Although these feeders were nearly of equal distance from the hive, the bees flew over similar ground (grassland), but in directions towards approximately east separated by an angle of 90°. The immediate surroundings of the two feeders were different; Feeder 1 was located in the open, close to a crossing path, and Feeder 2 next to bushes and to a small asphalt road. It is thus possible that the memory of the bees trained to feeder 1 and 2 differed with respect to landscape features to which they referred when exposed to the homing tests. In order to control for such effects the control groups and the treated groups were trained to both feeders in two sequential series of experiments (experiment 1 and 2).

A survival analysis was conducted on the data and a flight duration of 120 min was chosen for bees that did not come back to the hive as it was the time experimenters waited for them at the hive entrance. The flight duration of all other bees was the flight time in minutes from the release site to the hive.

**Fig 9:**
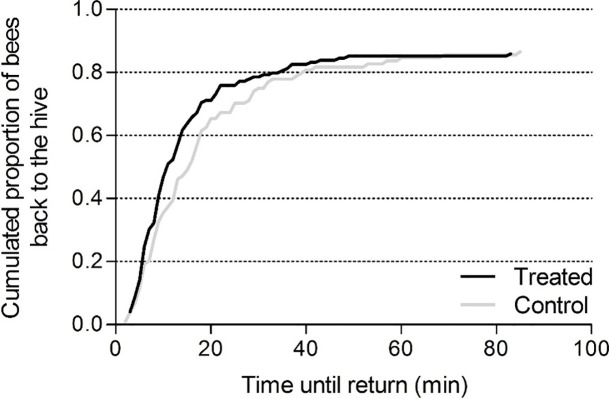
Probability of homing success as a function of time until return. Control and treated (4.5 and 9 ppb) honey bees returned to their hive in similar proportions (*n*_control_ = 104, 87% return; *n*_treated_ = 149, 86% return; Fisher's exact test, P = 1). The origin of the temporal axis represents the moment of release.

No difference was found in the homing success or flight duration of bees from the pre-exposure period (Table 1). No influence of the treatment was revealed on the homing success (Fig 9, control 87% return, treated, 87% return) when the experiment (feeder location) and concentrations were left out from the analysis and only the treatment effect was considered (Fig 9 and Table 1, 4.5 and 9 ppb together, Kaplan Meier Log Rank test χ_1_^2^ = 1.1, P = 0.29 and Fischer’s Exact test, P = 1). However, a significant difference was found in the flight duration of control and treated bees (Table 1, Mann Whitney, P < 0.05). Control bees took on average 3 minutes longer than bees exposed to 4.5 and 9 ppb clothianidin to return to the hive.

**Table 1:** Summary of the homing success and flight duration of honey bees released.

		Pre-exp.		Exp. 1		Exp. 1		Exp. 2		Total	
		no treatment		control	4.5 ppb	control	9 ppb	control	9 ppb	controls	4.5 + 9 ppb
feeder		F1	F2	F1	F2	F1	F2	F2	F1	F1+F2	F2+F1
*n* returned /*n* total		24/24	40/42	42/43	47/50	22/26	46/49	26/35	35/50	90/104	128/149
homing success (%) *		100	95	98	94	85	94	74	70	87	86
flight duration	Mean ± s.e.m. (min)	9.63 ± 1.53	9.43 ± 0.84	18.38 ± 2.47	9.94 ± 1.04	12.00 ± 1.80	14.37 ± 2.04	20.54± 3.36	16.80 ± 1.98	16.63 ± 2.08	13.41 ± 1.01
	median §	8	8.5	13	7	9	10	16	13	13	10
	min	3	3	2	3	3	3	8	3	2	3
	max	36	24	69	37	32	83	85	49	85	83

* Kaplan Meier Log Rank test (treatment) Total, χ_1_^2^ = 1.1, P = 0.29 (Fischer’s exact test, P = 1); (treatment + concentration + experiment): χ_5_^2^ = 34.9, P = 1.59e-6 followed by Tukey post-hoc tests, significant differences: control exp.1 *vs* control exp.2, P < 0.01; treated exp.1 *vs* treated exp.2, P < 0.001; control (4.5 ppb) *vs* control (9 ppb), P < 0.05; treated 4.5 ppb *vs* 9 ppb, P < 0.001; F1, 9 ppb, control *vs* treated, P < 0.05; F2, 9 ppb, control *vs* treated, P < 0.01.

§ Mann Whitney tests, significant differences: control *vs* treated 4.5 ppb, P < 0.01; Total, P < 0.05; control exp. 1 (9 ppb) *vs* control exp. 2 (9 ppb), P = 0.01; F2, 9 ppb, control *vs* treated, P < 0.05; F2, 9 ppb, control *vs* treated, P = 0.01.

A significant difference in the homing success was revealed for the experiment (the localization of the feeders) and the two different concentrations of clothianidin (4.5 and 9 ppb), (Table 2, Kaplan Meier Log Rank test treatment + concentration + experiment: χ_5_^2^ = 34.9, P < 0.001). The experiment (related to the feeding location, but also to weather conditions and colony status) had an influence on the homing success and the flight duration of the bees. Significant differences were revealed within the control group and within the treated group between bees foraging during experiment 1 or 2 (Tukey, treated, P < 0.001; control, P < 0.01) and between bees exposed to 4.5 or 9 ppb (Tukey, treated, P < 0.001; control, P < 0.05). Control bees foraging at F2 during experiment 2 had a lower homing success (Table 1, Tukey, P < 0.01) and flew significantly longer than treated bees foraging at F2 on 9 ppb during experiment 1 (Mann Whitney, P = 0.01). Bees exposed to 9 ppb clothianidin at the feeder F1 in experiment 2 had a lower homing success (Table 1, Tukey, P < 0.05) and flew significantly longer (Mann Whitney, P < 0.05) than control bees foraging at F1, but in experiment 1.

**Table 2:** Summary of the Cox regression model.

Variables	Model 1				Model 2			
	regr. coef	exp (coef) *	Z	P	regr. coef	exp (coef) *	Z	P
**treatment**	0.101	1.106	0.615	0.539				
**concentration**	-0.067	0.935	-0.244	0.807				
**experiment**	-0.837	0.433	-1.763	0.078	-0.478	0.620	-2.625	**0.009**
**time foraging ǂ**	-0.077	0.926	-1.912	0.056	-0.094	0.910	-2.692	**0.007**
**time exposure §**	0.029	1.030	0.784	0.433				
**temperature**	0.085	1.089	2.178	**0.029**	0.056	1.061	2.365	**0.018**
**time before flying $**	-0.046	0.955	-1.173	0.241	-0.056	0.946	-1.448	0.147
	*Rsquare*: *0*.*165 (max possible = 1)*, *Likelihood Ratio Test*: *45*.*62 on 7 df*, *P = 1*.*036e-7*				*Rsquare*: *0*.*162 (max possible = 1)*, *Likelihood Ratio Test*: *44*.*64 on 4 df*, *P = 4*.*727e-9*			

A backward selection on the AIC was performed on model 1 in order to obtain model 2

Values in bold indicate significant differences

*exp (coef) = Hazard ratio

ǂ **time foraging** is the time in days during which a bee is foraging at its feeder before being released

§ **time exposure** is the time in days from the first day of the experiment until the day the bee is released

$ **time before flying** is the short time bees waited at the release site before starting to fly

However, no significant difference in the homing success of control and treated bees was revealed in experiment 1 or experiment 2 (Table 1, Tukey, P = 0.12 and P = 0.94 respectively). In addition, no significant difference in the homing success was revealed between bees exposed to 4.5 ppb or 9 ppb and their relative controls (Table 1, Tukey, P = 0.08 and P = 0.5 respectively). The control bees that returned to the hive in experiment 1 flew significantly longer than the treated bees exposed to 4.5 ppb in experiment 1 (Table 1, Mann Whitney, P < 0.01).

The influence of multiple variables on the homing success was tested in a cox-regression model. We found no effect of the treatment and of the concentration (4.5 or 9 ppb) on honey bee homing success (Table 2, model 1). In a reduced model, the experiment and the time foraging had a significant influence on the homing success (Table 2, model 2) whereas the time of exposure and time before flying had no impact.

Also, the temperature at the release time had a significant effect on honey bee homing abilities. Indeed, the temperature was lower during the second part of the experiments (average temperature exp. 1 = 21.7°C, exp. 2 = 18.4°C), which could have influenced the homing success [51].

Based on the crop-emptying measurements by Fournier *et al*. [52] it was calculated that the foragers could have assimilated in 50 min up to about 8 μl of the sucrose solution collected at the treated feeder, corresponding to 0.04 ng (4.5 ppb exposure) and 0.08 ng (9 ppb exposure) clothianidin respectively. This amount was what bees would take up just before flying, in addition to the residues already assimilated over *n* days foraging at the feeder. This value is a higher estimate because the amount of assimilated sucrose during the 50 min waiting time may well be much lower depending on the activity of the waiting bee [53]. In any case the partial acute treatment component involved in the homing success experiments adds to the chronic effect.

## 4. Discussion

The effects of a neonicotinoid insecticide, clothianidin, under chronic intoxication in field conditions was investigated and an impairment of the normal foraging behavior and feeder visitation rates was revealed. These effects may result from impaired memory processing and memory recall as substantiated in laboratory experiments [15, 42].

Several parameters need to be considered when studying pharmacological effects on animal behavior at sublethal doses, in particular whether the substance is detected orally by its taste or smell and whether it impairs the life span of the animal. Kessler *et al*. [54] found that none of the concentrations of clothianidin tested (greater than in our experiments), altered the spiking activity of sucrose sensitive gustatory neurons in the bumblebees’ or the honeybees’ sensillae. Another study [42] indicated that bees do not prefer or avoid clothianidin when tested for their sucrose responsiveness or when put in a choice situation in semi-field conditions. If there is no sensory discrimination, it is thus likely that bees should not be able to prefer or avoid a contaminated crop.

The results of the survival analysis showed that clothianidin and its concentration (4.5 or 9 ppb) had no significant negative effect on honey bee homing success (Table 2), however the consumption of clothianidin-contaminated nectar led to reduced foraging efficiency and recruitment rates (Figs 2 and 4). Choice was made to pool the 4.5 ppb and 9 ppb concentrations for the analysis of the foraging span because a large majority of bees that started to forage on 9 ppb were previously exposed to 4.5 ppb and vice versa. At the beginning of the experiment, bees could not be clearly sorted out between the two concentrations. Foraging behavior may have differed between the two concentrations but we believe that omitting those bees in the analysis would have introduced a bigger bias due to low sample size and minimized the effects on the foragers and the colony.

We found that honey bees visiting a feeder containing clothianidin foraged over shorter periods of time, probably because they died earlier than the control bees. This result is not surprising as other studies already reported the same effects with other neonicotinoids [14, 55]. Also, morphological and histochemical alterations were observed in the brain structures and midgut from exposed bees, contributing to the reduction of their lifespan [55]. Furthermore, the overexpression of the vitellogenin transcript in the brains of exposed honey bees could explain the alteration in foraging activity and accelerated aging [56].

The number of days a bee foraged at its feeder before being released had a significant effect on the homing success, indicating that the duration of the exposure to clothianidin and thus the accumulated doses mattered. Furthermore, the experiment and thus the duration of the experiment (related to weather conditions and status of the colony) and/or the feeding location had an influence on the homing success and the flight duration. Because these bees foraged at different feeding locations, the effect indicates a training site-specific component. Also, the temperature at the release time had a significant effect on honey bee homing abilities. The temperature was lower during the second half of the summer, which could have influenced the homing success of bees. Henry *et al*. [51] showed such an influence of the weather and temperature in their study with thiamethoxam.

Bees foraging chronically at a feeder contaminated with 4.5 ppb or 9 ppb clothianidin collected on average about 13 ng or 14 ng (experiment 1 and experiment 2, respectively) clothianidin per bee and per day (S1 Table). These estimations based on our measurements correspond to the EFSA [37] estimations of how much residues a honey bee can collect when foraging on oilseed rape treated with the highest maximal application rate of clothianidin (up to 13.65 ng per forager bee in one day). A forager could collect between 0.28 ng and 0.53 ng of clothianidin on one trip at the feeder depending on the experiment and the concentration of the clothianidin solution at the feeder. Schneider *et al*. [13] determined a significant reduction (31%) of the number of feeder visits per bee compared to the control group at a dosis of 0.5 ng/bee. In our experiment with chronic uptake of similar or lower amounts of clothianidin at the feeder (9 ppb) led to a reduction of the foraging activity at the contaminated feeder (Fig 3, 33% in experiment 1 and 34% in experiment 2). It is most likely that bees remained inside the hive until the acute spiking effect of clothianidin ceased and they were motivated to fly out to the feeder again [13].

A prolonged stay inside the hive was probably not used for dance communication because activity at the feeder was highly affected by a chronic uptake of clothianidin (Fig 3), as was already shown with imidacloprid [10] and thiacloprid [14]. Even higher concentrations of sucrose at the contaminated feeder could not totally compensate for the reduced dance activity during the dance-induction periods. The results on regular foraging activity and dance performance show that clothianidin most likely alters the motivation to forage rather than the sensory or motor components of foraging. A reduced visitation of flowers by bees would impair pollination efficiency in the long-term [3] leading to dramatic consequence on honey bees, biodiversity and agriculture.

Three different behaviors inside of the hive were distinguished with electrostatic field measurements, fanning, waggle dancing and stop signaling. Bees engage in fanning when controlling temperature, humidity and CO_2_ concentration. Fanning occurs particularly intensively during processing nectar. The fanning signals are likely to act also as social signals communicating the amount of additional energy necessary to produce honey from the collected nectar [57]. Waggle dancing and stop signaling are tightly connected. Stop signals produced by dance followers induces trophallaxis with the dancer [45] and thus adds to the communication process in waggle dancing. Stop signals are also produced by bees that aim to terminate dance performance e.g. by a scout bee during nest site communication [58] and between food foragers when an adverse situation has been detected at the food site [59]. Reduced activities in all three signals indicates a profound impairment of social communication by clothianidin, a result similar to what was found for thiacloprid [14].

Contrary to the results of our previous field study with thiacloprid [14], no impairment in honey bees homing rates was revealed in this study (Fig 9). Schneider *et al*. [13] found that 100% of the control and 94.4% of the treated bees returned to the hive during a three-hour observation period immediately after an acute treatment with 0.5 ng clothianidin. Based on the crop-emptying measurements by Fournier *et al*. [52] the foragers in our experiment could have assimilated in 50 min 0.04 ng to 0.08 ng clothianidin depending on the concentration at the feeder. This very low “acute” dose (adding up to the chronic exposure) might simply not be sufficient to impair the homing success and navigation of bees. On the contrary to thiacloprid which seem to accumulate also in the bee heads, exposure to clothianidin led to an accumulation only in the abdomens of the foragers (Fig 8, S2 Table). The fact that clothianidin was mostly found in the abdomens and not much in the heads where the nAChR are located could explain why the homing success of these bees was not impaired.

This study explored a large range of behaviors in field conditions and all behaviors related to foraging or social communication were found significantly impaired by a chronic exposure to clothianidin at sublethal concentration. The foraging span, the foraging activity, the number of waggle runs, the fanning time, and the number of stop signals were significantly lower in the exposed colony. Furthermore, the residues of clothianidin were found to accumulate in the abdomens of exposed foraging bees. However, no difference was found in the homing success and the flight duration between control and treated bees.

Although only a single control and a single treatment colony were used, several signals appear strong and this study sets the stage for future investigations, especially on social signals described with electrostatical field measurements which could be used for large-scale monitoring of honey bee colonies’ health.

## Supporting information

S1 Table Sucrose consumption at the feeders and estimated amounts of clothianidin collected. (DOCX)

S2 Table Pesticide residues analysis of honey bees chronically exposed to clothianidin in the field. (DOCX)

S3 Table Details about the LC-MS/MS method used. (DOCX)
