# Supplementary material for: Detrimental effects of clothianidin on foraging and dance communication in honey bees
Source: PLoS One. 2020 Oct 29;15(10):e0241134. doi: 10.1371/journal.pone.0241134 (PMC7595294; doi:10.1371/journal.pone.0241134)
Supplement: S1 Table — (DOCX) [file pone.0241134.s001.docx]

**S1 Table:** **Sucrose consumption at the feeders and estimated amounts of clothianidin collected.**

|  | duration feeder open (min) | total consump  -tion /day (ml) | No. of bees at the feeder | estimated sucrose collected /bee /day (ml) | estimated clothianidin collected /bee  /day (ng) | | estimated No. of trips /bee /day |
| --- | --- | --- | --- | --- | --- | --- | --- |
| 4.5 ppb, experiment 1 | | | | | | | |
| control | 457.71  ± 17.54 | 113.86  ± 10.02 | 41 ± 1 | 2.75  ± 0.24 |  | 55.26  ± 3.46 | |
| treated | 463.14  ± 16.65 | 127.29  ± 3.73 | 50 ± 1 | 2.59  ± 0.09 | 12.95  ± 0.45 | 45.60  ± 6.12 | |
| 9 ppb, experiment 1 | | | | | | | |
| control | 401.00  ± 44.89 | 113.00  ± 14.00 | 37 ± 3 | 3.00  ± 0.14 |  | 43.93  ± 3.27 | |
| treated | 421.86  ± 47.25 | 106.57  ± 15.26 | 40 ± 4 | 2.58  ± 0.24 | 12.90  ± 1.21 | 28.88  ± 2.78 | |
| 9 ppb, experiment 2 | | | | | | | |
| control | 416.36  ± 9.86 | 71.05  ± 5.39 | 38 ± 2 | 1.91  ± 0.16 |  | 46.17  ± 3.82 | |
| treated | 384.27  ± 15.96 | 52.73  ± 5.30 | 27 ± 2 | 1.43  ± 0.16 | 14.32  ± 1.62 | 27.03  ± 2.21 | |

Numbers shown are means ± s.e.m.
