# Supplementary material for: Detrimental effects of clothianidin on foraging and dance communication in honey bees
Source: PLoS One. 2020 Oct 29;15(10):e0241134. doi: 10.1371/journal.pone.0241134 (PMC7595294; doi:10.1371/journal.pone.0241134)
Supplement: S2 Table — (DOCX) [file pone.0241134.s002.docx]

**S2 Table. Pesticide residues analysis of honey bees chronically exposed to clothianidin in the field**

|  | days of exposure  * | sample weight (mg)  # | clothianidin residues (ng/g) | | | | (ng/bee) |
| --- | --- | --- | --- | --- | --- | --- | --- |
|  |  |  | head | thorax | abdomen | **whole body** | **whole body** |
| **Feeder**  **Exp. 1** | 1-2 days | 894.5 | n.d. | n.d. | 2.2 | **2.2** | **0.2** |
|  | 3-6 days | 1249.5 | n.d. | n.d. | 2.1 | **2.1** | **0.2** |
|  | 7-+ days | 1051.3 | n.d. | n.d. | 2.9 | **2.9** | **0.3** |
| **Feeder**  **Exp. 2** | 1-2 days | 982.7 | n.d. | n.d. | 2.4 | **2.4** | **0.2** |
|  | 3-6 days | 954.0 | 2.8 | n.d. | n.d. | **2.8** | **0.3** |
|  | 7-+ days | 1002.3 | n.d. | n.d. | 3.2 | **3.2** | **0.3** |
| **Hive** | 7 days | 764.1 | n.d. | n.d. | n.d. | **n.d.** | **n.d.** |
|  | 30 days | 829.8 | n.d. | n.d. | 2.8 | **2.8** | **0.2** |
| ***LOD*** *§* | |  | *1.2* | *0.4* | *0.5* |  |  |
| ***LOQ*** *§* | |  | *2.4* | *0.8* | *1.2* |  |  |
| ***Recovery (% RSD)*** | |  | *81 % (5.5 %)* | *87.6 % (7 %)* | *104.8 % (10 %)* |  |  |

No residues were detected in any of the control samples analyzed

* Days of exposure Feeders: number of days foraging at the feeders before sample collection / Hive: number of days since the beginning of the experiment

# The sample weight is the sum of the weights of the separated analyzed honeybee body parts (from 9 to 13 bees/sample)

n.d. not detectable

§ LOD, limit of detection (3 times background noise); LOQ, limit of quantification (10 times background noise). The calculation is based on an average weight of 10 bee body parts each.
