# Supplementary material for: Detrimental effects of clothianidin on foraging and dance communication in honey bees
Source: PLoS One. 2020 Oct 29;15(10):e0241134. doi: 10.1371/journal.pone.0241134 (PMC7595294; doi:10.1371/journal.pone.0241134)
Supplement: S3 Table — (DOCX) [file pone.0241134.s003.docx]

**S3 Table. Details about the LC-MS/MS method used**

| LIQUID CHROMATOGRAPHY UltiMate 3000 RS (Dionex) | | | |
| --- | --- | --- | --- |
| Autosampler temperature | 10 °C | | |
| Injection volume | 2 µL | | |
| Syringe rinse | 100 µL | | |
| Analytical column | Phenomenex Kinetex C18 (2.6 µm, 100 mm, 3 mm i.d.) | | |
| Column temperature | 60 °C | | |
| Mobile phase A | Methanol + 0.1 % acetic acid + 1 mM ammonium acetate | | |
| Mobile phase B | Water + 0.1 % acetic acid + 1 mM ammonium acetate | | |
| Gradient program | Time (min) | B (%) | A (%) |
|  | 0 | 98 | 2 |
|  | 1 | 2 | 98 |
|  | 3 | 2 | 98 |
|  | 3 | 98 | 2 |
|  | 4 | 98 | 2 |
| Flow rate | 500 µL/min | | |
| MASS SPECTROMETER | QTRAP 5500 (AB SCIEX)  Analyst 1.6.1 | | |
| Mode | positive ESI | | |

| Q1 Mass | Q3 Mass | Dwell time | Analyte | DP | EP | CE | CXP |
| --- | --- | --- | --- | --- | --- | --- | --- |
| Positive mode | | | | | | | |
| 253 | 172 | 50 | Clothianidin-d3 | 42 | 10 | 19 | 14 |
| 226 | 126 | 50 | Acetamiprid-d3 | 33 | 10 | 29 | 11 |
| 250 | 169 | 50 | Clothianidin 1 | 42 | 10 | 19 | 12 |
| 250 | 132 | 50 | Clothianidin 2 | 42 | 10 | 35 | 10 |
